# Phylogenetic Constraints and Environmental Filtering Jointly Drive Adaptive Evolution in *Phragmites australis* : From Genetic Structure to Trait Decoupling on the Mongolian Plateau

**DOI:** 10.1002/ece3.74332

**Published:** 2026-09-06

**Authors:** Rui Zhang, Huamin Liu, Shan Jiang, Zhichao Xu, Yunhao Wen, Zhenhua Dang, Lu Wen, Lixin Wang

**Affiliations:** ^1^ Ministry of Education Key Laboratory of Ecology and Resource Use of the Mongolian Plateau, Inner Mongolia Key Laboratory of River and Lake Ecology, School of Ecology and Environment Inner Mongolia University Hohhot China; ^2^ School of Geography and Planning Jining Normal University Ulanqab China

**Keywords:** evolutionary decoupling, Mongolia Plateau, *Phragmites australis*, phylogenetic niche conservatism, plant functional traits

## Abstract

The Mongolian Plateau, a typical arid and semi‐arid zone in Eurasia, is characterized by highly heterogeneous and fragmented wetland habitats. *Phragmites australis* , a common wetland species in this region, exhibits remarkable adaptability. Unraveling the coordination between phylogenetic history and local environmental filtering is crucial for elucidating its adaptive mechanisms. Integrating landscape genomics and trait‐based phylogenetic analyses, we analyzed transcriptome‐wide SNPs, multidimensional functional traits, and environmental variables across 90 individuals from 30 natural *P. australis* populations. This study aims to reveal the genetic and phenotypic variation patterns underlying population genetic structure and trait variation, specifically distinguishing the roles of geographic isolation, environmental filtering, and phylogenetic history. Results reveal a significant drainage‐dependent pattern in genetic structure. Populations in hydrologically connected basins show extensive admixture, whereas those in isolated endorheic basins form distinct lineages. While geographic isolation underpins genetic differentiation, environmental filtering independently explains ~33.84% of the genetic variation, driven primarily by moisture heterogeneity (precipitation seasonality and soil moisture). Crucially, we observed differentiated evolutionary trajectories across functional traits. Structural traits (e.g., plant height, leaf thickness) are phylogenetically conserved; in contrast, physiological traits (e.g., water use efficiency) are decoupled from phylogeny, showing patterns consistent with high plasticity regulated by local environments. This evolutionary decoupling strategy enables *P. australis* to flexibly adapt to heterogeneous habitats while maintaining structural stability. This study uncovers the synergistic mechanisms by which geographic isolation and environmental filtering jointly shape the genetic patterns of this cosmopolitan species at a regional scale, clarifies that its evolutionary responses may depend heavily on the differentiated plasticity of trait types, and provides valuable regional insights into how widespread wetland species adapt to heterogeneous environments under global change.

## Graphical abstract

Integrating landscape genomics and functional traits across the Mongolian Plateau, we reveal that *Phragmites australis* exhibits a genetic structure shaped by hydrological connectivity and moisture heterogeneity. Crucially, we identify a strategy of evolutionary decoupling where physiological traits remain highly plastic to adapt to local environments, while structural traits remain phylogenetically conserved to ensure stability.

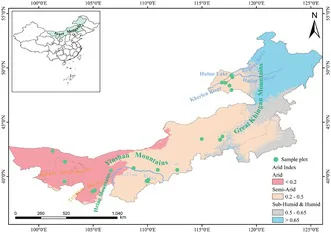

## 1 Introduction

The spatial distribution and adaptive patterns of species in heterogeneous habitats are intrinsically governed by the interaction between historical evolutionary processes and contemporary ecological contexts (Orsini et al. 2013). The population genetic structure of cosmopolitan species is typically the result of the joint interplay between neutral differentiation caused by dispersal limitation and local adaptation driven by environmental filtering (Sexton et al. 2014). The classic “Isolation by Distance” (IBD) model emphasizes the restrictive role of geographical barriers on gene flow (Wright 1943), whereas the “Isolation by Environment” (IBE) model posits that strong habitat heterogeneity is sufficient to maintain specific adaptive genotypes even in the presence of gene flow (Wang et al. 2013; Wang and Bradburd 2014). However, the extent to which local selective pressure can override the spatial constraints imposed by geographic isolation to shape independent, genome‐wide adaptive variation patterns remains a core issue to be elucidated in evolutionary ecology (Savolainen et al. 2013; Yeaman 2022).

In real‐world geographical landscapes, these two forces are not mutually exclusive. Instead, they jointly shape the genetic patterns of populations (Liu et al. 2023; Lazic et al. 2024). Functional traits serve as a bridge connecting genotypes to the environment. Furthermore, they represent the direct manifestation through which natural selection acts upon individuals (Violle et al. 2007; Caruso et al. 2017). Adaptive signals at the genomic level must ultimately be translated into individual fitness through phenotypic variation (Flood and Hancock 2017). During adaptive evolution, heterogeneous habitats exert directional selective pressures. These pressures filter for trait combinations with specific functional attributes, thereby driving phenotypic differentiation (Stark et al. 2017; Richards et al. 2019). Although constrained by evolutionary history, key functional traits that sensitively respond to environmental gradients can often form phenotypic landscapes that match environmental variations across populations (Oliveras et al. 2020). Crucially, the significant spatial congruence between habitat‐driven phenotypic differentiation and genomic variation suggests that IBE shapes population genetic structure independently of IBD. This pattern reveals adaptive differentiation at the genomic level (Rellstab et al. 2015). Therefore, it is essential to dissect how key functional traits respond to specific environmental drivers and to evaluate the intrinsic link between phenotypic differentiation and genetic structure. Such analysis is key to verifying whether local selective pressures can reshape population genetic structure and to revealing how cosmopolitan species such as *P. australis* achieve regional adaptation in heterogeneous landscapes.

The phenotypic responses of plants to the environment are simultaneously constrained by their long evolutionary history (Wiens et al. 2010). According to the Phylogenetic Niche Conservatism (PNC) hypothesis, species tend to retain ancestral trait characteristics. This phylogenetic inertia may limit their potential to adapt to new environments, resulting in trait variation that exhibits significant phylogenetic signals rather than environmental associations (Losos 2008). This presents an evolutionary “paradox”: environmental selection drives trait divergence, whereas ancestral constraints tend to limit trait divergence. To address this dilemma, the concept of “Mosaic Evolution” suggests that different types of traits within an organism may follow independent evolutionary trajectories (Ackerly 2009; Opedal et al. 2022). Relevant studies indicate that structural traits related to fundamental plant architecture and mechanical support (e.g., plant height, anatomical structures) may be highly conservative due to strong phylogenetic constraints. Conversely, physiological traits directly involved in resource acquisition and metabolic regulation may be decoupled from phylogenetic constraints, exhibiting patterns consistent with high plasticity in response to the environment, although the relative contribution of genetic differentiation remains to be tested (Ávila‐Lovera et al. 2023; Zhao et al. 2026). This differentiated trait response mechanism may be a key strategy for cosmopolitan species to successfully occupy highly heterogeneous habitats while maintaining the stability of their basic body plan. However, few studies have systematically verified this by integrating population genetic structure with multidimensional phenotypic data.

As a typical arid and semi‐arid ecological zone in the hinterland of Eurasia, the Mongolian Plateau features a highly fragmented and significantly heterogeneous wetland landscape, which has been jointly shaped by historical hydrological changes and continuous climatic fluctuations (Xu et al. 2019; Jie et al. 2021). This unique geographical configuration provides an ideal natural laboratory for verifying the aforementioned genetic and phenotypic evolutionary mechanisms. *Phragmites australis* , the dominant species in the wetland ecosystems of this region, exhibits extremely high phenotypic plasticity and a broad ecological niche (Kettenring et al. 2011; Jia et al. 2024). Although previous studies have focused on its morphological variation, a deep understanding of how this species copes with multiple environmental pressures by coordinating the stability of its genetic structure with the flexibility of its functional traits is still lacking (Eller et al. 2017; Song et al. 2024; Wei et al. 2025).

Integrating transcriptome‐wide single‐nucleotide polymorphism (SNP) data, environmental factors, and multidimensional functional traits, this study utilizes landscape genomic approaches and phylogenetic signal and constraint analyses to elucidate the adaptive evolutionary mechanisms of *P. australis* populations on the Mongolian Plateau. Based on niche theory and adaptive evolution hypotheses, we propose the following scientific hypotheses: (1) Although geographic isolation resulting from historical drainage system changes may dominate the overall genetic structure (IBD pattern), we hypothesize that the heterogeneous environment still exerts sufficient selective pressure to drive significant adaptive genetic differentiation (IBE pattern) after controlling for geographic distance. (2) Regarding response strategies to heterogeneous habitats, we hypothesize that the phenotypic evolution of *P. australis* may exhibit evolutionary decoupling among traits. Specifically, we expect that not all traits display identical rates of divergence; instead, they display differentiated response patterns: certain fundamental structural traits related to the species' constitution may retain strong phylogenetic signal, reflecting developmental and structural constraints that limit rapid morphological change; conversely, other traits closely associated with resource acquisition and stress resistance may exhibit higher environmental sensitivity and are primarily driven by the filtering of local habitat factors (Ávila‐Lovera et al. 2023).

## 2 Method

### 2.1 Study Area and Sample Collection

**FIGURE 1:**
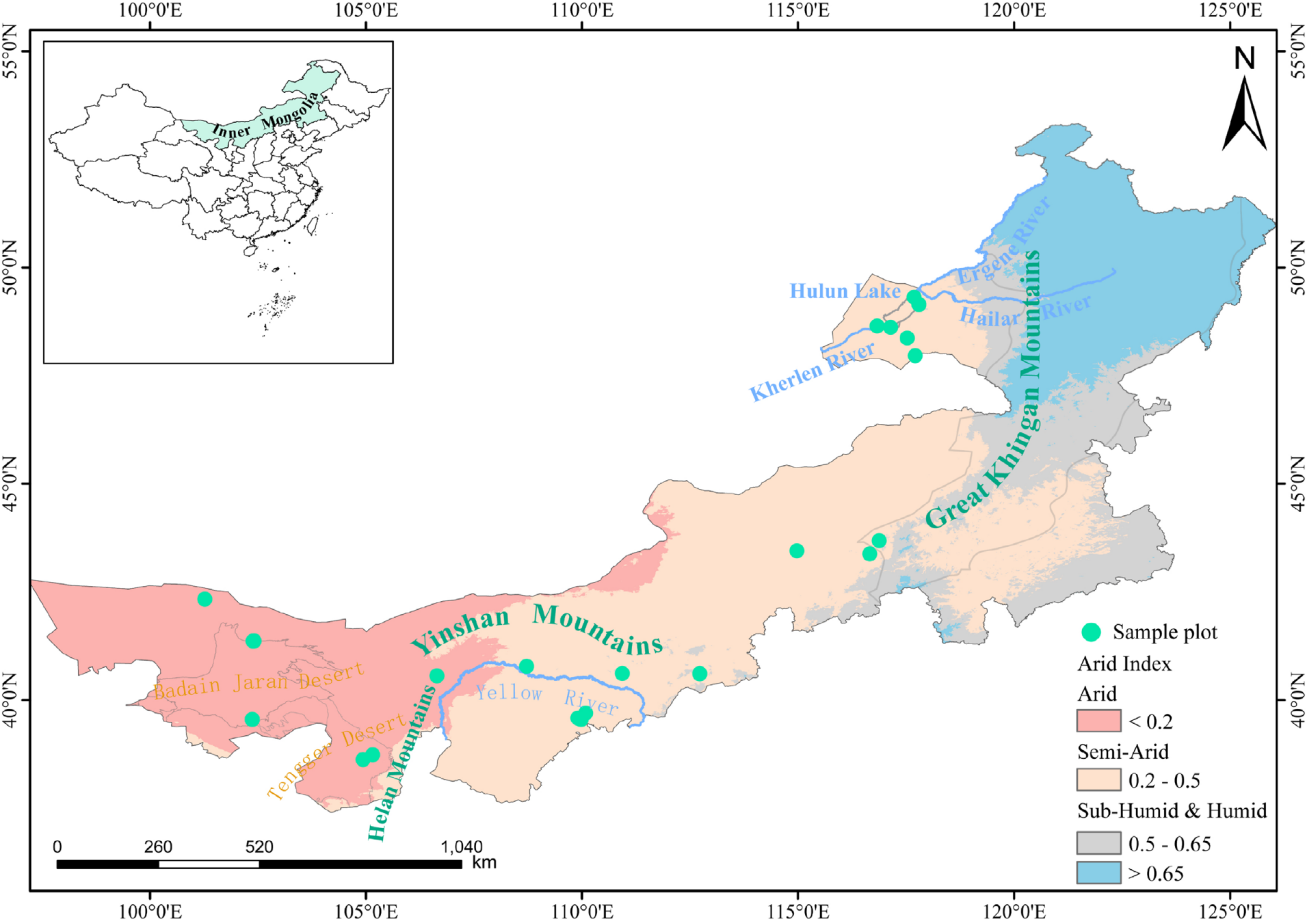
Spatial geographic distribution of *P. australis* sampling populations along the aridity gradient on the Mongolian Plateau. Solid green circles indicate the geographical coordinates of the sampling plots. The background color gradient represents the Aridity Index (AI), classified into climatic zones: arid (AI < 0.2), semi‐arid (AI: 0.2–0.5), and sub‐humid and humid (AI > 0.5).

This study was conducted on the Mongolian Plateau, a typical arid and semi‐arid biogeographical region. The region's complex history of hydrological evolution and highly fragmented wetland landscapes provided an ideal natural laboratory for exploring the local adaptation of cosmopolitan species. During the peak growing season in July 2023, we selected 30 representative natural populations of *P. australis* with minimal anthropogenic disturbance along the regional aridity gradient (Figure 1; Table S1). The sampling sites covered diverse habitat types, including lacustrine wetlands, marshes, and saline meadows. These sites encompass a continuous macro‐climatic gradient of temperature and precipitation across arid and semi‐arid zones, while localized hydrological conditions across these diverse micro‐habitats further generate distinct variation in local environmental conditions.

Within each population, five healthy, pest‐free mature individuals were randomly selected. Their plant height and photosynthetic parameters were measured in situ, and leaves were collected for subsequent morphological and anatomical analyses. Plant samples were transported to the laboratory in portable coolers. Three individuals were randomly sub‐sampled from these five measured plants per population for transcriptomic sequencing analysis (yielding 90 individuals in total across 30 populations). To minimize genetic similarity arising from clonal growth, the sampling distance between individuals was maintained at greater than 10 m. Healthy apical leaves from these sub‐sampled individuals were harvested, immediately flash‐frozen in liquid nitrogen in the field, and subsequently stored in a −80°C ultra‐low temperature freezer for transcriptomic sequencing analysis.

### 2.2 Functional Trait Measurement

This study measured a series of key functional traits related to resource acquisition, nutrient utilization, photosynthetic physiology, and anatomical structure (Table 1). Trait measurements were performed on the selected mature individuals. To standardize leaf sampling, the second fully expanded functional leaf from the plant apex was consistently collected for the measurement of all parameters. Morphological traits were assessed using standard digital analysis protocols. Leaf samples were scanned, and their morphological parameters were quantified using image analysis software. Photosynthetic parameters were measured in the field on sunny mornings using a portable photosynthesis system (LI‐6800, LI‐COR) under controlled environmental conditions: leaf chamber CO_2_ concentration was maintained at 400 μmol·mol^−1^, leaf chamber H_2_O concentration at ~20 mmol·mol^−1^, and photosynthetically active radiation at a saturating light intensity of 1500 μmol·m^−2^·s^−1^. Data were recorded after stomatal conductance and photosynthetic rate stabilized.

**TABLE 1:** Functional traits and abbreviations of *P. australis* .

Type & parameter	Abbreviations	Ecological significance	References
Plant height	H	Competitive ability for light and vertical space occupation strategy	Poorter et al. (2012)
Leaf thickness	LT	Resource storage capacity and physical defense mechanism	Poorter et al. (2009)
Specific leaf area	SLA	Resource acquisition efficiency and “fast investment‐return” strategy	Wright et al. (2004)
Leaf dry matter content	LDMC	Tissue density construction, physical toughness, and resource conservation	Poorter et al. (2009)
Leaf carbon content	LCC	Structural carbon investment cost and tissue construction basis	Poorter et al. (2012)
Leaf nitrogen content	LNC	Photosynthetic capacity and enzymatic investment basis	Evans (1989), Wright et al. (2004)
Net photosynthetic rate	A	Instantaneous carbon assimilation capacity and potential growth rate	Liang et al. (2020)
Transpiration rate	E	Water transport, nutrient uptake, and leaf temperature regulation	Liang et al. (2020)
Water use efficiency	WUE	Carbon‐water trade‐off strategy and drought adaptability	Hatfield and Dold (2019)
Leaf epidermis thickness	LET	Barrier against water loss and a buffer mechanism for temperature fluctuation	Ni et al. (2022)
Leaf upper epidermis to lower epidermis ratio	UE/LE	Structural balance between transpiration regulation and photoprotection	Ni et al. (2022)
Leaf xylem diameter	LXD	Leaf water transport efficiency and hydraulic supply–demand balance	Sack and Scoffoni (2013)
Leaf phloem diameter	LPD	Photosynthate export capacity and source‐sink coordination	Sack and Scoffoni (2013)
Leaf vascular bundle area	LVBA	Synergy between transport capacity and mechanical support	Sack and Scoffoni (2013)

To assess anatomical characteristics, fresh tissue samples were collected from healthy, intact plants. Leaf samples (8 mm × 8 mm) were excised from the middle one‐third of the leaf (Sack and Scoffoni 2013). Tissues were immediately fixed in FAA (Formalin‐Aceto‐Alcohol) fixative in the field and stored at low temperature. In the laboratory, fixed tissues were treated with 10% glycerol, vacuum‐penetrated with OCT embedding medium, and frozen at −80°C. Frozen sections of 8–10 μm thickness were cut using a cryostat microtome, mounted on adhesive slides, and set on a slide warmer at 37°C for 3 min. After washing 3–5 times with PBS buffer (5 min each) to remove OCT, sections were stained with Safranin‐Solid Green for 3–5 min, washed with distilled water, and mounted with 20% glycerol. Transverse sections were stained and imaged under a light microscope (Ruzin 1999). Anatomical structures of leaf tissues were measured using ImageJ 1.54 software (Figure S1) (Rasband 2017). To ensure data accuracy, five measurements were taken for each anatomical section (technical replicates) to calculate the mean value for each individual. Five such individuals (biological replicates) were assessed per population (Guo et al. 2008; Lynch et al. 2021). A complete list of measured traits and their ecological significance is presented in Table 1, and detailed measurement protocols are provided in Table S2.

### 2.3 Environmental Factor Measurement

Soil cores were collected from the 0–30 cm depth profile. At each sampling point, three soil cores were taken near the plant root system and combined to form one representative composite soil sample. Within each population, three such independent replicate soil samples were collected. Soil moisture (SM, %) was determined gravimetrically by weighing fresh samples, oven‐drying them at 105°C for 48 h, and reweighing.

The remaining soil samples were air‐dried and sieved for physicochemical analyses. Soil pH was measured in a 1:10 (w/v) soil–water suspension, and electrical conductivity (EC, μS/cm)—an indicator of soil salinity—was measured in a 1:5 (w/v) soil–water extract (Ismayilov et al. 2021). Total soil carbon (TC, mg/g) and nitrogen (TN, mg/g) concentrations were quantified using an elemental analyzer (Vario EL, Elementar, Germany) (Gembal and Gałązka 2013).

Climatic variables for each site were extracted from the WorldClim 2.0 database and the Global Aridity Index and Potential Evapotranspiration Database (version 3) based on geographic coordinates (Zomer et al. 2022). Key bioclimatic variables included mean annual temperature (MAT), mean diurnal range (DDT), mean annual evapotranspiration (MAE), mean annual precipitation (AP), and seasonal precipitation variability (SVP). The Aridity Index (AI) was used to classify the climatic context of sampling sites, ranging from arid (AI < 0.2) to semi‐arid (0.2 ≤ AI < 0.5) and sub‐humid/humid (AI > 0.5) zones (Fick and Hijmans 2017).

### 2.4 SNP Identification and Quality Control

Total RNA was extracted from leaf tissue using a modified CTAB method (Aboul‐Maaty and Oraby 2019), and 150 bp paired‐end sequencing was performed on the DNBSEQ‐T7 platform. Raw sequencing reads were first processed for quality control using Fastp (v0.23.4) to trim low‐quality bases (Phred score < 20) and remove adapter sequences. Clean paired‐end reads were subsequently mapped to the *P. australis* reference genome (GenBank: GCA_040373225.1) using STAR (v2.7.10a) with splice alignment and strand‐specific options enabled (Dobin et al. 2013; Wang et al. 2024).

Following alignment, SAMtools was used for file conversion, sorting, and indexing. Picard Tools was applied to add read group information (AddOrReplaceReadGroups) and mark PCR duplicates (MarkDuplicates). To account for intron‐exon spliced reads in RNA‐seq data, mapped reads were further processed using GATK (v4.6.1.0)'s SplitNCigarReads to split reads spanning splicing events. Variant calling was then performed independently for each sample using HaplotypeCaller to generate individual VCF files, which were subsequently merged into a population‐level raw VCF file using GatherVcfs. Bi‐allelic SNP variants were extracted using SelectVariants (McKenna et al. 2010; DePristo et al. 2011).

To ensure the reliability of the genomic dataset, preliminary hard filtering of raw SNPs was performed using GATK's VariantFiltration tool with parameters optimized for RNA‐seq: QD < 2.0, FS > 30.0, and MQ < 40.0. Subsequently, VCFtools (v0.1.17) was used to remove Indels and low‐quality variants, retaining only SNPs with a quality score (*Q*) ≥ 30, mean depth (DP) ≥ 5, missing rate ≤ 20%, and minor allele frequency (MAF) ≥ 0.05 (Danecek et al. 2011). For population structure and phylogenetic analyses, we further performed linkage disequilibrium (LD) pruning using PLINK (Chang et al. 2015) with parameters set to ‐‐indep‐pairwise 50 5 0.2. This step aimed to eliminate bias caused by tightly linked loci, thereby obtaining a highly independent SNP set for subsequent evolutionary analyses.

### 2.5 Population Genetic Analysis

Based on the quality‐controlled SNP dataset, we evaluated the genetic composition of *P. australis* populations using Admixture (v1.3.0) software (Alexander et al. 2009) by running clustering models from *K* = 1 to *K* = 10 and determining the optimal number of clusters based on the cross‐validation error (CV error).

Subsequently, Principal Component Analysis (PCA) was performed using PLINK to visually display the genetic differentiation patterns of populations from different geographic regions in a multidimensional space. To quantify intra‐population genetic characteristics, we used VCFtools (Danecek et al. 2011) to calculate nucleotide diversity, observed heterozygosity, and expected heterozygosity for each sampling site and estimated the inter‐population fixation index (*F*_st_) to assess the degree of genetic differentiation.

To infer the phylogenetic relationships among populations, high‐quality, LD‐pruned SNP data in VCF format were converted into a concatenated multiple sequence alignment file in PHYLIP format using the Python script vcf2phylip (Ortiz 2019). The resulting alignment matrix preserved all polymorphic loci across all individuals. Maximum Likelihood phylogenetic trees were constructed using IQ‐TREE2 (v2.3.6) (Minh et al. 2020). The optimal substitution model was automatically selected using the built‐in ModelFinder (Kalyaanamoorthy et al. 2017), and branch reliability was evaluated via 1000 ultrafast bootstrap replicates (Ultrafast Bootstrap, ‐bb 1000) to ensure the accuracy of the topology.

### 2.6 Statistical

Geographic distance matrices between individuals were calculated using the geosphere (Karney 2013). To evaluate the distribution of genetic variation among and within populations, Analysis of Molecular Variance (AMOVA) was performed using the poppr package (Kamvar et al. 2014). Statistical significance for variance components and Fixation index (Φ) statistics (ΦST [Overall among all populations], ΦSC [Among populations within regions], and ΦCT [Among regions]) was evaluated using 999 random permutations. Mantel and partial Mantel tests (Dixon 2003) were performed to assess correlations between genetic distance and environmental, phenotypic, and geographic distances, controlling for geographic distance to extract independent effects. Multiple Matrix Regression with Randomization (MMRR) was employed to incorporate the aforementioned distance matrices, quantifying the relative contributions of IBD and IBE to genetic variation. To deeply analyze the genetic structure, Principal Coordinate Analysis (PCoA) was performed on genetic distances. Variance Partitioning Analysis (VPA) was then used to decompose total genetic variation into fractions explained by geography, environment, and their joint effects. Concurrently, partial canonical ordination (partial RDA) (Davies and Tso 1982) combined with forward selection (Blanchet et al. 2008) was utilized to identify significant environmental drivers of genetic structure and to evaluate their importance.

To evaluate the phylogenetic conservatism of phenotypic traits in *P. australis* populations on the Mongolian Plateau and their adaptive relationships with environmental factors, we conducted phylogenetic signal analysis and Phylogenetic Generalized Least Squares (PGLS) analysis. Based on the constructed phylogenetic tree, Blomberg's *K* and Pagel's *λ* statistics for 14 phenotypic traits were calculated using phytools (v2.5‐2) (Blomberg et al. 2003) to assess trait clustering on the phylogeny. Simultaneously, Mantel tests (Dixon 2003) were conducted between pairwise phenotypic distance and genetic distance matrices to evaluate whether phenotypic divergence among populations is proportional to their overall genetic distance. PGLS models based on maximum likelihood estimation were employed to analyze the effects of environmental factors on phenotypic traits (Tung Ho and Ané 2014), effectively controlling for phylogenetic relatedness among populations. The strength of associations was evaluated via standardized regression coefficients and coefficients of determination (*R* ^2^), with False Discovery Rate (FDR) correction applied to infer statistical significance in multiple testing.

To further parse the relative contributions of different environmental factor groups to phenotypic variation, hierarchical partitioning analysis was performed. Environmental factors were categorized into five groups: Soil Nutrients (TN, TC), Soil Salinity and Alkalinity (EC, pH), Soil Moisture (SM), Climatic Heat (MTA, DDT), and Climatic Moisture (AP, SVP, AI). Subsequently, the independent and joint contributions of each group were calculated using phylolm.hp (Lai et al. 2025) to quantify their relative importance in explaining phenotypic variation. All statistical analyses were performed in R v4.3.2 (R Core Team 2021).

## 3 Result

### 3.1 Characteristics of Genetic Variation in *P. australis* Populations on the Mongolian Plateau

**FIGURE 2:**
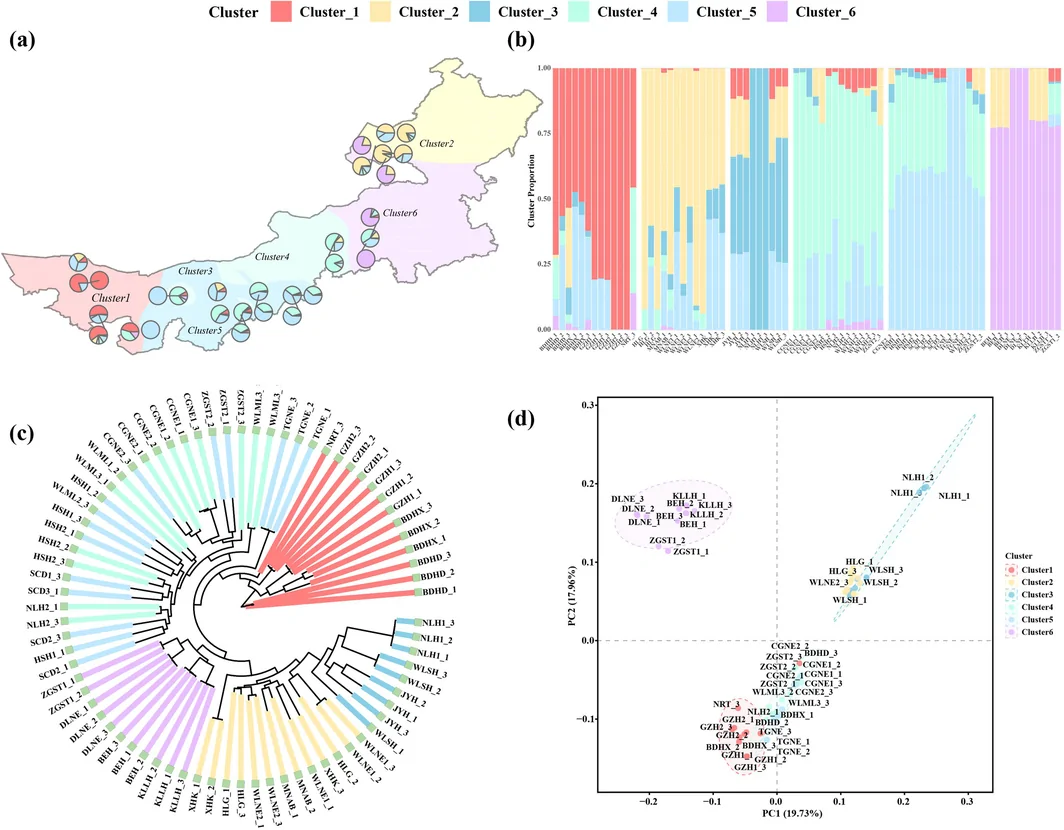
Characteristics of genetic variation in *P. australis* populations across the Mongolian Plateau. (a) Spatial distribution of genetic components in Mongolian Plateau *P. australis* populations, showing the geographical locations of sampling sites and the population genetic composition at *K* = 6. The proportional areas of different colors within each pie chart represent the average ancestral composition of the six genetic clusters within that population. (b) ADMIXTURE bar plot for Mongolian Plateau *P. australis* populations. Each vertical bar represents an individual sample, with different colors indicating the six inferred ancestral genetic components. (c) Maximum Likelihood (ML) phylogenetic tree of Mongolian Plateau *P. australis* populations. (d) Principal Component Analysis (PCA) scatter plot of the genetic structure of Mongolian Plateau *P. australis* populations. Each point represents an individual sample, colored according to its assigned genetic cluster. The dashed ellipses indicate the 95% confidence intervals for the individuals within each cluster.

In total, 727,255 high‐quality SNPs were obtained from the transcriptome data. After linkage disequilibrium pruning, 175,012 independent SNPs were retained for evolutionary analysis. Population structure analysis (ADMIXTURE) combined with cross‐validation error evaluation (optimal *K* = 6, Figure S2) indicated that *P. australis* populations on the Mongolian Plateau could be divided into six major genetic clusters (Clusters 1–6), exhibiting a significant geographical clustering pattern (Figure 2a,b). Among them, populations in geographically isolated desert lakes and endorheic basins (landlocked hydrological systems that retain water and have no outflow to external bodies such as oceans; for example, Cluster 1, mainly distributed within deserts) possessed relatively simple genetic components, reflecting strong genetic independence. Cluster 3, located in the transitional zone between Western and Central Inner Mongolia, displayed a highly mixed and heterogeneous genetic composition. In contrast, the central wetland network (e.g., Clusters 4 and 5) and the eastern wetland network (e.g., Clusters 2 and 6), which have high watershed connectivity, showed obvious multi‐source genetic admixture, suggesting continuous or intermittent gene flow among internal lakes and rivers (Figures 1 and 2). PCA further demonstrated that the samples displayed a continuous distribution pattern in the ordination space that highly matched their geographical span (Figure 2d). The Maximum Likelihood tree constructed based on the optimal model (GTR + F + ASC + R4) was highly consistent with the population structure analysis (Figure 2c; Table S3). Notably, populations from desert lakes and endorheic basins exhibited longer branch lengths, implying a history of long‐term independent evolution. This suggests that geographic isolation may be the dominant force driving the evolutionary history of these *P. australis* populations.

Analysis of genetic diversity results showed that genetic diversity across all genetic clusters of *P. australis* populations on the Mongolian Plateau was maintained at a moderate level (He ranged from 0.105 to 0.222). A general phenomenon of heterozygote excess (inbreeding coefficient *F* values were all negative) was observed (Table S4). Analysis of Molecular Variance (AMOVA) indicated that genetic variation mainly originated from within populations (71.53%), while the contribution of variation among populations within regions was 12.71%, consistently indicating a moderate level of genetic differentiation among populations (Table S5). Pairwise *F*_st_ analysis further revealed that the differentiation was most significant between the geographically isolated Cluster 3 and Cluster 6 (*F*_st_ = 0.157), while it was weakest between the environmentally similar and spatially continuous Cluster 4 and Cluster 5 (*F*_st_ = 0.061). At the population level, pairwise *F*_st_ exhibited a broad distribution ranging from 0 to 0.595, with an overall mean of 0.062 (Figure S3). This clarifies that within the context of overall moderate differentiation, significant geographic lineage divergence exists among *P. australis* populations on the Mongolian Plateau (Figure S4).

### 3.2 Geographic and Environmental Drivers of Genetic Differentiation in *P. australis* Populations on the Mongolian Plateau

Mantel tests showed that genetic distance was significantly positively correlated with geographic distance, environmental distance, and phenotypic distance (*p* < 0.001), indicating that all three are associated with the distribution pattern of the genetic structure of *P. australis* populations (Table S6). MMRR results further confirmed that the independent contribution of each factor reached a highly significant level (*p* < 0.001). Standardized regression coefficients showed that geographic distance made the largest contribution (*β* = 0.315), followed by environmental distance (*β* = 0.117), clarifying that geographic isolation plays a significant role in the genetic differentiation of *P. australis* populations.

VPA results indicated that environmental factors and geographic distance jointly explained 55.15% of the genetic variation. When controlling for spatial distance, environmental factors independently accounted for 33.84% of the variation. In contrast, geographic distance alone explained only 4.45% of the variation after controlling for environmental variables. The joint variation explained by both components was 16.86%, reflecting a moderate spatial autocorrelation between environmental conditions and geographic distance. The remaining 44.85% of unexplained variation may stem from unmeasured ecological drivers, historical demographics, or micro‐environmental noise (Figure S5). This suggests that while geographic isolation plays a foundational role in population genetic differentiation, environmental selective pressure also plays a prominent role in shaping the genetic structure of *P. australis* populations on the Mongolian Plateau.

**FIGURE 3:**
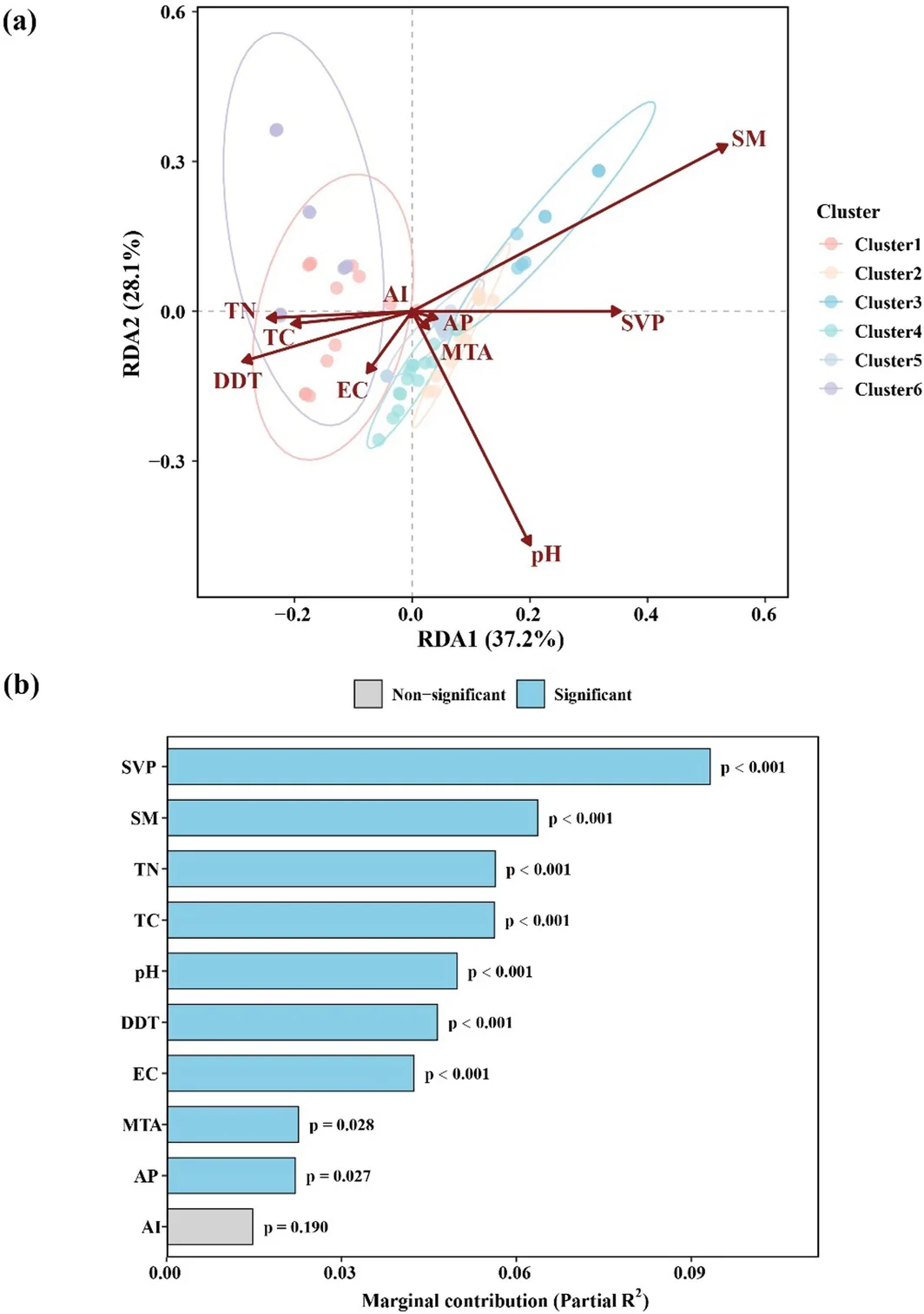
Relationship between genetic variation and environmental factors in *P. australis* populations across the Mongolian Plateau based on Partial Canonical Ordination (partial RDA). The analysis assesses the effect of environmental factors on genetic variation after controlling for geographical distance as a covariate. (a) Partial RDA ordination plot: point colors correspond to genetic clusters; reddish‐brown arrow vectors represent environmental factors. (b) Partial *R* ^2^ (marginal contribution) of each environmental factor, with blue bars indicating factors that are statistically significant (*p* < 0.05).

After controlling for the effects of geographic spatial autocorrelation, partial RDA results showed that environmental factors had significant independent explanatory power for the genetic structure of *P. australis* (Figure 3a). The first two RDA axes cumulatively explained 45.3% of the total variation, with the RDA1 axis (27.2%) mainly driven by SVP, SM, TC, DDT, and TN; while the RDA2 axis (18.1%) showed a high correlation with soil pH, SM, and EC. Concurrently, we clarified the impact weights of different environmental variables on genetic patterns; SVP had the highest independent explanatory rate (9.32%, *p* < 0.001), followed by SM (6.36%, *p* < 0.001), indicating that moisture heterogeneity is the primary environmental factor affecting the genetic structure of populations in this region. In addition, TN (5.64%), TC (5.62%), and soil pH (4.98%) also showed significant independent contributions, illustrating that habitat physicochemical properties also have a significant impact on genetic variation (Figure 3b).

### 3.3 Phylogenetic Signal and Ecological Adaptation Analysis of Phenotypic Traits in *P. australis* Populations on the Mongolian Plateau

Phylogenetic signal analysis indicated that the phenotypic traits of *P. australis* populations on the Mongolian Plateau exhibited differentiated evolutionary patterns (Table 2). H and LT showed the strongest phylogenetic conservatism; their Blomberg's *K*, Pagel's *λ*, and trait‐genetic distance correlations (Mantel *r*) all reached highly significant levels (*p* < 0.001), indicating that these traits are strongly constrained by phylogenetic history. Although traits such as SLA, LDMC, and A showed significant Blomberg's *K* and Pagel's *λ*, their Mantel tests with genetic distance were not significant. This suggests that their variation, while structured by phylogenetic history, is also shaped by environmental selection. Furthermore, LCC, LNC, LXD, and LVBA exhibited weak phylogenetic signals, reflecting lower evolutionary conservatism.

**TABLE 2:** Phylogenetic signal of phenotypic traits in *P. australis* populations from the Mongolian Plateau.

Trait	Blomberg's *K*	*K p*‐value	Pagel's *λ*	*λ p*‐value	Mantel *r*	Mantel *p*‐value
H	0.807	< 0.001	0.993	< 0.001	0.305	< 0.001
LT	0.415	< 0.001	0.740	< 0.001	0.197	< 0.001
SLA	0.444	0.019	0.972	< 0.001	−0.082	0.884
LDMC	0.437	0.003	0.902	< 0.001	0.076	0.071
LCC	0.351	0.165	0.773	< 0.001	0.007	0.457
LNC	0.360	0.127	0.788	< 0.001	0.002	0.493
A	0.439	0.005	0.848	< 0.001	0.064	0.113
E	0.384	0.056	0.331	0.039	0.124	0.128
WUE	0.358	0.094	0.293	0.005	0.031	0.304
UE.LE	0.455	0.007	0.932	< 0.001	−0.073	0.893
LET	0.419	0.011	0.831	< 0.001	−0.105	0.964
LXD	0.374	0.059	0.731	0.002	0.047	0.205
LPD	0.393	0.032	0.812	< 0.001	−0.028	0.698
LVBA	0.364	0.064	0.562	< 0.001	0.023	0.317

*Note:* Blomberg's *K* and Pagel's *λ* measure the conservatism of traits across the phylogenetic tree; *K p*‐value and *λ p*‐value represent significance test values; Mantel *r* denotes the correlation coefficient between trait distance and genetic distance.

**FIGURE 4:**
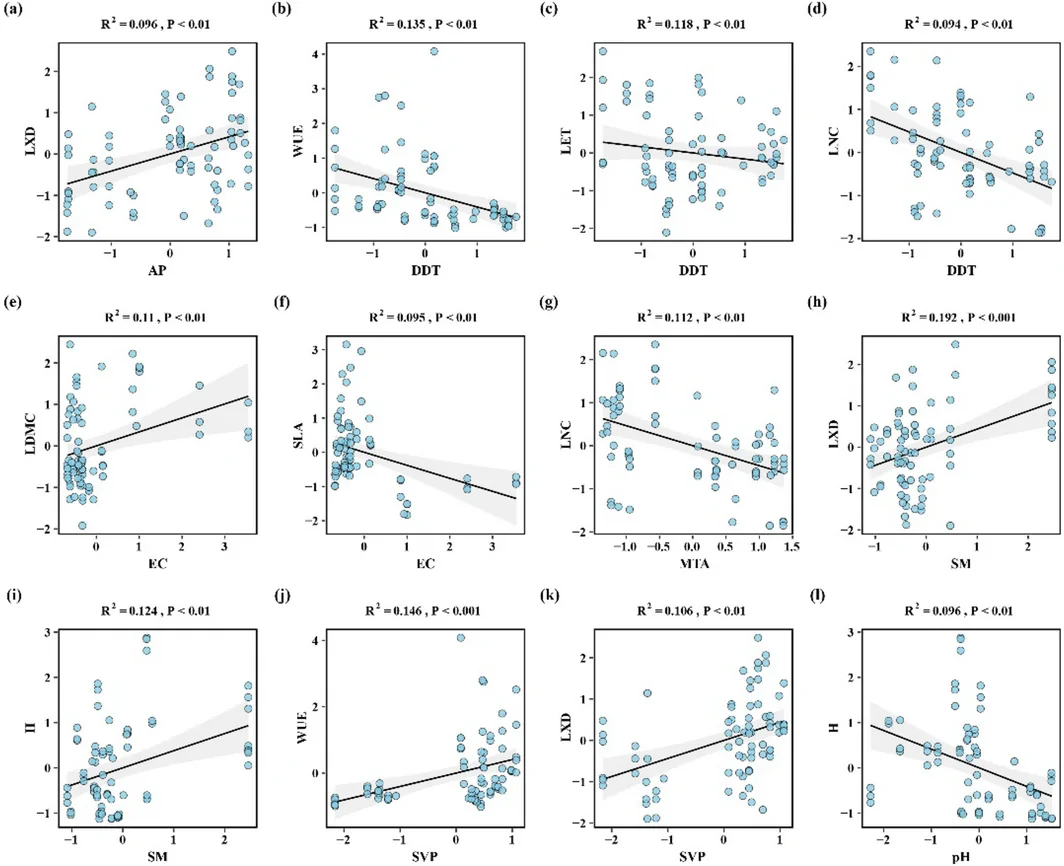
Significant phylogenetic generalized least squares (PGLS) relationships between phenotypic traits and environmental factors in *P. australis* populations from the Mongolian Plateau. The fitting results show the variation of phenotypic traits along environmental gradients after controlling for the phylogenetic relatedness among populations. Blue scatter points represent observed values, the solid line indicates the regression line fitted by the PGLS model, and the gray shaded area denotes the 95% confidence interval. The adjusted coefficient of determination (*R* ^2^) and the significance *p*‐value for each model are provided in the figure.

After controlling for phylogenetic relationships, environmental factors exerted significant and differentiated driving effects on the phenotypic traits of *P. australis* (Figure 4). WUE was significantly positively correlated with SVP and significantly negatively correlated with DDT; meanwhile, LXD was mainly positively driven by SM. Regarding leaf resource allocation strategies, LNC significantly decreased with increasing MTA and DDT. Additionally, habitat salinization drove a shift in *P. australis* leaves from a resource‐acquisition strategy to a conservative strategy. This shift was manifested as a significant increase in leaf construction costs: specifically, as EC increased, SLA significantly decreased, accompanied by a synergistic increase in LDMC. Temperature also affected leaf physical defense, with LET showing a significant negative correlation with DDT.

**FIGURE 5:**
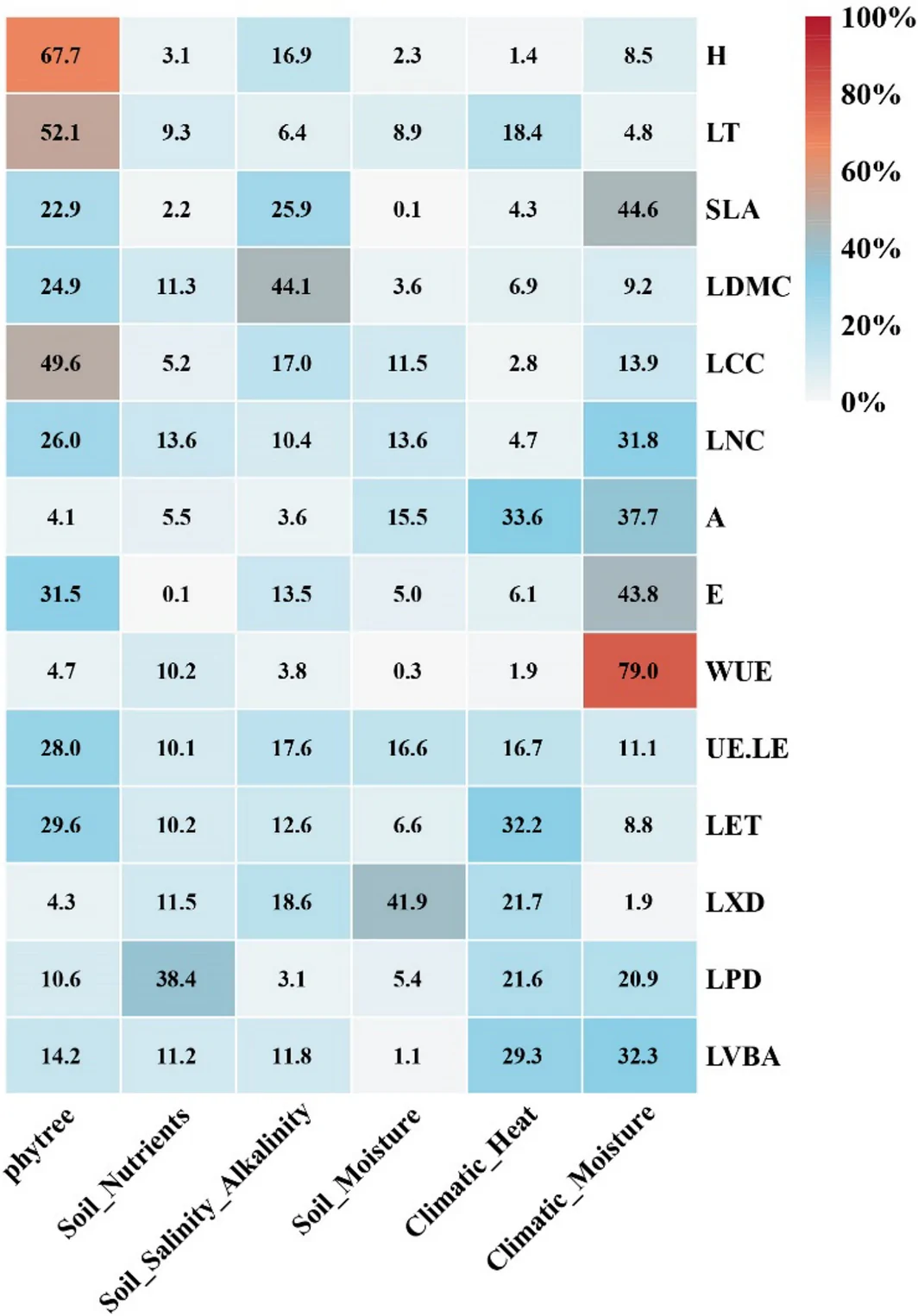
Hierarchical partitioning analysis of phenotypic trait variation in Mongolian Plateau *P. australis* populations: relative contributions of environmental factor groups and phylogeny. The horizontal axis represents different explanatory sources: phytree (phylogenetic signal); Soil_Nutrients (TN, TC); Soil_Salinity_Alkalinity (pH, EC); Soil_Moisture (SM); Climatic_Heat (MTA, DDT); Climatic_Moisture (AP, SVP, AI). Heatmap values represent the percentage of independent contribution, with a color gradient from white to red indicating increasing contribution magnitude.

Hierarchical partitioning analysis quantitatively decomposed the relative contributions of phylogenetic constraints and multidimensional environmental factors to phenotypic variation (Figure 5). The analysis revealed that structural traits were mainly controlled by genetic background, with the variation in H (67.7%), LT (52.1%), and LCC (49.6%) largely explained by phylogenetic history. In contrast, physiological traits were mainly driven by local habitat filtering; in particular, WUE (79.0%), SLA (44.6%), and E (43.8%) exhibited extremely strong dependence on climatic moisture. Moreover, different environmental factors showed distinct trait‐specific contributions: soil salinity and alkalinity and moisture conditions dominated the variation in LDMC (44.1%) and LXD (41.9%), respectively, while soil nutrients mainly influenced transport tissue development (LPD, 38.4%). Climatic heat factors played a key role in regulating photosynthetic performance (A, 33.6%) and leaf defense functions (LET, 32.2%).

## 4 Discussion

### 4.1 The Joint Shaping of Genetic Patterns by Hydro‐Geographic Patterns and Environmental Filtering

The genetic distribution of *P. australis* populations on the Mongolian Plateau exhibits a typical “water system‐dependent” pattern. This phylogeographic structure, which overlaps highly with watershed and lake networks, profoundly reflects the constraints imposed by historical hydrological processes on the gene flow of wetland plants (Nilsson et al. 2010; Patamsytė et al. 2023). In China, *P. australis* is broadly structured into northwestern tetraploid (4×) lineages associated with cpDNA haplotypes O and M, as well as eastern octoploid (8×) lineages associated with haplotype P (An et al. 2012; Liu et al. 2022). Against this phylogeographic backdrop, populations located in endorheic regions or desert hinterlands (such as Cluster 1) possess relatively simple and unique genetic components. This strong geographic isolation is primarily driven by the vast expanses of unsuitable, hyper‐arid terrestrial matrix (e.g., the Badain Jaran and Tengger deserts) intervening between isolated water bodies, which lack continuous hydrological corridors and severely restrict the long‐distance dispersal of both pollen and seeds. Related studies suggest that these isolated groups could potentially represent conserved ancestral tetraploid lineages adapted to extreme hyper‐arid conditions (An et al. 2012). Consequently, isolated populations have formed distinct evolutionary units under the influence of genetic drift and differential selection pressures (Orsini et al. 2013; Qiu et al. 2023).

In contrast, populations distributed in major river basins and ecotones between water systems exhibit high levels of genetic admixture. This phenomenon may reflect secondary contact and potential interploidy introgression where western tetraploid and eastern octoploid lineages overlap along regional hydrological corridors (Liu et al. 2022). Hydrological connectivity acts as a “corridor” for gene flow, effectively mitigating isolation effects, which is consistent with previous findings in wetland plants (Werth and Scheidegger 2014; Liu et al. 2021). This difference in genetic structure determined by water system connectivity makes landscape features play a fundamental role in shaping the genetic patterns of wetland plant populations.

Additionally, our results showed a consistent pattern of heterozygote excess (*F* < 0) across all sampled populations. Extensive vegetative propagation via rhizomes allows *P. australis* populations to bypass sexual recombination. This clonal reproduction effectively preserves heterozygous genomic combinations over time (Balloux et al. 2003; Halkett et al. 2005). Furthermore, polyploidy plays a critical role in maintaining genetic diversity. Due to polysomic inheritance and homeologous gene duplications, polyploidy inherently promotes higher individual heterozygosity (Meirmans et al. 2018). In our study, Cluster 6 likely represents the eastern octoploid lineage (haplotype P). Geographically, its distribution aligns with the primary range of haplotype P (Liu et al. 2022). Moreover, Cluster 6 is genetically distinct from other populations, a differentiation that most likely reflects the underlying ploidy differences between tetraploid and octoploid cytotypes in northern China. Importantly, the remarkably high heterozygosity observed in this cluster is highly consistent with the elevated genetic diversity typically accompanying higher ploidy levels (Meirmans and Van Tienderen 2013). We speculate that the persistence of clonal lineages, together with multi‐copy genomic structures, jointly facilitates the maintenance of high genomic heterozygosity in these wetland populations.

Isolation by Distance remains the dominant force shaping the overall genetic pattern of *P. australis* on the Mongolian Plateau. This aligns with findings in many widely distributed plant systems, which posit that on a broad geographical scale, dispersal limitation is a primary factor leading to genetic differentiation (Sexton et al. 2014). However, going beyond mere geographic isolation, environmental filtering exhibits significant plasticity at the micro‐evolutionary scale (Liu et al. 2023). Although geographic distance limits the dispersal of neutral genes, heterogeneous habitats (especially moisture and salinity gradients) constitute a more rigorous evolutionary mechanism. In arid and semi‐arid regions, strong environmental selective pressure drives significant adaptive differentiation at the genetic level in local populations (Orsini et al. 2013). This process might be further modulated by cytotype‐specific physiological tolerances, such as differential drought and salinity endurance between tetraploids and octoploids (Liu et al. 2022). This implies that *P. australis* populations are not merely passively constrained by geographic dispersal limitations; instead, through genetic responses consistently associated with specific habitat patches, they exhibit genetic variation patterns that reflect local environmental heterogeneity. This accumulation of independent variation driven by the environment suggests that niche specialization is becoming a core driving force for this species to adapt to heterogeneous habitats (Morris et al. 2011).

### 4.2 Evolutionary Decoupling of Traits: Interplay Between Historical Lineage Constraints and Environmental Selection

The successful colonization of *P. australis* populations in the heterogeneous habitats of the Mongolian Plateau did not rely on the concerted evolution of all traits. Instead, it exhibited significant characteristics of “Mosaic Evolution”, where traits with different functional attributes follow differentiated evolutionary trajectories (DiFrisco 2023). Our study confirms that structural traits related to plant architecture and physiological traits responsible for resource acquisition have significantly decoupled in their evolutionary trajectories. This divergence reflects a complex trade‐off by plants between biophysical limitations and evolutionary historical constraints under multidimensional environmental pressures. Structural traits (e.g., H, LT) constitute the fundamental mechanical framework of the plant body. Their construction involves cell wall synthesis and the stable development of vascular tissues, thus being subject to strict allometric relationships and biomechanical constraints (Niklas and Telewski 2022). These intrinsic physical constraints predispose structural traits to maintain evolutionary stability, ensuring basic hydraulic safety and mechanical support in environments with strong winds or water deficits (Pierce et al. 2022). This aligns with the Phylogenetic Niche Conservatism hypothesis, suggesting that this morphological conservatism in *P. australis* reflects genetic stability formed over long‐term evolution, limiting its potential for substantial variation in response to short‐term environmental fluctuations (Ávila‐Lovera et al. 2023; Cruz‐Nicolás et al. 2024). In sharp contrast to highly conserved structural traits, physiological functional traits directly involved in resource acquisition and metabolic regulation (e.g., WUE, LNC) exhibit significant evolutionary decoupling from phylogenetic history. Consistent with this, comparative studies across diverse plant clades have shown that structural traits (e.g., leaf thickness and anatomy) generally exhibit stronger phylogenetic signals than physiological traits (e.g., WUE and photosynthetic rate), which tend to be more evolutionarily labile and environmentally driven (Ávila‐Lovera et al. 2023). These traits are decoupled from phylogenetic constraints, are primarily controlled by local environmental factors, and exhibit trait variation patterns consistent with high phenotypic plasticity. This is highly consistent with the well‐documented ecophysiological flexibility in *P. australis* , whose photosynthetic machinery, gas exchange capacity, and photophysiological traits exhibit remarkable plasticity in response to fluctuating temperature, moisture, and microclimatic stresses across diverse geographical regions (Eller et al. 2017). Such inherent photosynthetic and physiological flexibility allows populations to rapidly acclimate their metabolic performance to local environmental variations, enabling cosmopolitan species like *P. australis* to maximize resource acquisition while maintaining basic morphological stability through this evolutionary strategy of “conservatism in structural traits versus divergence in physiological traits”. This asynchronous trait evolutionary mechanism may be the key reason why cosmopolitan species can still occupy broad ecological niches and achieve local adaptation even in the absence of significant morphological differentiation.

Concurrently, we found that in arid and semi‐arid regions, the physiological traits of *P. australis* exhibited distinct responses to precipitation. The tight coupling between water use efficiency and seasonal precipitation reveals the species' carbon assimilation strategy. Maximizing carbon gain through physiological regulation during the precipitation‐concentrated growing season represents a convergent adaptation to environments with high evaporative demand (Huang et al. 2025). *P. australis* exhibits shifts in photosynthetic pathways along the C3–C4 continuum under drought stress. Studies have shown that in arid environments, the upregulation of C4‐like metabolism, characterized by increased phosphoenolpyruvate carboxylase activity and higher leaf δ^13^C values, enhances carbon fixation efficiency per unit of water loss. This provides a biochemical basis for this physiological flexibility without requiring structural evolutionary changes (Zheng et al. 2000; Zhu et al. 2012). In saline‐alkali stress habitats, *P. australis* demonstrates an “Ecological Strategy Shift” from “fast investment‐return” to “slow investment‐high tolerance.” High‐salinity environments drive leaf traits toward a “conservative” strategy, characterized by decreased SLA and increased LDMC. Although this phenotypic variation increases leaf construction costs, it significantly enhances tissue tolerance to ion toxicity and physiological drought, aligning with theoretical predictions of the Leaf Economics Spectrum in stressful environments (Joswig et al. 2022). Notably, although traits such as SLA still retain certain phylogenetic traces, environmental filtering has evidently become the dominant force at the local scale, driving adaptive phenotypic differentiation within populations. Furthermore, the significant association between xylem vessel diameter and soil moisture further confirms the adaptive mechanism by which *P. australis* balances water transport efficiency and embolism risk by regulating its hydraulic structure (Ávila‐Lovera et al. 2023). This multidimensional physiological plasticity and resource strategy adjustment collectively constitute the physiological basis for *P. australis* adaptation to the heterogeneous environment of the Mongolian Plateau.

By organically integrating landscape genomics with phylogenetic signal and trait‐constraint analyses, this study constructed a multidimensional analytical framework of “environment‐genetics‐traits,” revealing how cosmopolitan species achieve an evolutionary balance historical constraints and environmentally driven phenotypic plasticity. This finding not only deepens our understanding of plant adaptive evolutionary mechanisms but also provides a new perspective for predicting the evolutionary responses of cosmopolitan species in the context of global change. This study still has certain methodological limitations. Regarding the selection of genetic markers, we employed SNP markers developed based on transcriptome sequencing (RNA‐Seq). Compared to Whole Genome Sequencing (WGS), this strategy cannot cover genetic variation in non‐coding regulatory sequences such as introns and intergenic regions, potentially missing the contribution of certain regulatory elements to adaptation. However, the transcriptome SNP strategy, which focuses on expressed regions, can capture coding region variations directly related to function in a more cost‐effective manner (Moragues et al. 2010; Mascher et al. 2013; Takahagi et al. 2016). This enables us to more accurately identify functional gene loci subjected to directional environmental selection. Future research integrating whole‐genome data with common garden experiments would help to more comprehensively dissect the relative contributions of genetic variation and phenotypic plasticity to local adaptation, revealing the molecular basis of plant adaptation at the level of gene regulatory networks. Furthermore, it should be acknowledged that our field‐based sampling design cannot unequivocally distinguish between genetically based local adaptation and phenotypic plasticity in driving physiological trait variation (e.g., WUE). The observed decoupling and environmental correlations are consistent with high plasticity, but reciprocal transplant or controlled common garden experiments will be required to rigorously quantify their relative contributions.

## 5 Conclusion

By integrating population genetics and phylogenetic comparative analyses, this study reveals the spatial genetic differentiation and trait variation patterns of *P. australis* populations on the Mongolian Plateau, elucidating the synergistic roles of geographic isolation, environmental filtering, and phylogenetic history in shaping the adaptive patterns of this cosmopolitan species. The genetic structure of *P. australis* populations exhibits a significant drainage‐dependent pattern: basins with strong hydrological connectivity maintain high levels of gene flow and genetic admixture, whereas populations in isolated endorheic lake basins form distinct genetic lineages due to dispersal limitations; although geographic isolation serves as the foundational framework for genetic differentiation, the role of environmental filtering is equally significant. In arid and semi‐arid environments, strong local environmental selective pressures are sufficient to drive adaptive differentiation at the genomic level. Crucially, different functional traits follow differentiated evolutionary trajectories. Structural traits such as plant height and leaf thickness are subject to strong phylogenetic constraints and exhibit significant conservatism; in contrast, physiological traits such as water use efficiency and photosynthesis‐related traits are decoupled from phylogenetic history, being primarily regulated by current environmental factors and displaying patterns consistent with high phenotypic plasticity. This evolutionary strategy enables *P. australis* to effectively adapt to the heterogeneous and variable wetland habitats of the Mongolian Plateau. This study not only deepens our understanding of the spatial genetic differentiation and trait variation patterns in *P. australis* but also suggests that, in the context of future climate and land‐use changes, the evolutionary response of widespread wetland plants in arid/semi‐arid regions may depend heavily on the differentiated plasticity of trait types. These findings provide valuable regional insights for predicting and managing the evolutionary dynamics of wetland plants in changing environments.

## Author Contributions

**Rui Zhang:** conceptualization (equal), data curation (lead), formal analysis (lead), investigation (lead), methodology (equal), visualization (lead), writing – original draft (lead), writing – review and editing (equal). **Huamin Liu:** investigation (supporting), writing – review and editing (supporting). **Shan Jiang:** investigation (supporting), writing – review and editing (supporting). **Zhichao Xu:** investigation (supporting), writing – review and editing (supporting). **Yunhao Wen:** investigation (supporting), writing – review and editing (supporting). **Zhenhua Dang:** conceptualization (equal), project administration (equal), supervision (equal), writing – review and editing (equal). **Lu Wen:** investigation (supporting), writing – review and editing (supporting). **Lixin Wang:** conceptualization (equal), funding acquisition (lead), methodology (equal), project administration (lead), supervision (equal), writing – review and editing (equal).

## Funding

This work was supported by the National Natural Science Funds, P.R. China (No. 32571847, 32460288), Guiding Funds of Central Government for Supporting the Development of the Local Science and Technology (2024ZY0128), CSPON Project, China (No. 2023ZRBSHZ064), and the Science and Technology Major Project of Inner Mongolia (2025YFHH0185).

## Conflicts of Interest

The authors declare no conflicts of interest.

## Supporting information


**Data S1:** Supporting Information.

**Table S1:** Geographic and climatic characteristics of the 30 *Phragmites australis* study sites: Mean Annual Precipitation (P), Mean Annual Temperature (T), and Coordinates of the Plot.
**Table S2:** Description of functional traits, units, and detailed measurement protocols.
**Table S3:** Model selection parameters for phylogenetic trees (optimal model selected based on the lowest AICc value).
**Table S4:** Genetic diversity indices of *P. australis* populations from different geographic groups in the Mongolian Plateau.
**Table S5:** Φ‐Statistics of population genetic differentiation.
**Table S6:** Results of correlation analysis between genetic distance and geographical, environmental, and phenotypic distances.
**Figure S1:** Anatomical cross‐sections of *P. australis* leaf. (a, b) Leaf cross‐sections showing the epidermis and vascular bundles (xylem and phloem).
**Figure S2:** Inference graph for selecting the ancestral population number (*K*‐value) of the *P. australis* populations in the Mongolian Plateau. Based on cross‐entropy evaluation results from the Sparse Non‐negative Matrix Factorization (SNMF) algorithm, the horizontal axis represents the assumed number of ancestral populations (*K*‐value ranging from 1 to 10), while the vertical axis indicates the corresponding cross‐entropy score. The scatter points reflect the model goodness‐of‐fit across different *K*‐values, with lower cross‐entropy values suggesting a more optimal population subdivision scheme.
**Figure S3:** Frequency distribution of pairwise genetic differentiation coefficient (*F*_st_) among *P. australis* populations in the Mongolian Plateau. The histogram displays the distribution of *F*_st_ values calculated between all pairs of sampling populations. The horizontal axis represents the calculated *F*_st_ value, and the vertical axis represents the frequency (Count). The text above the graph indicates the mean pairwise *F*_st_ value for all samples.
**Figure S4:** Heatmap of pairwise *F*_st_ differentiation among the six major genetic groups of *P. australis* populations in the Mongolian Plateau. The matrix illustrates the genetic differentiation between different genetic groups (Cluster 1–6). The values within the grids represent pairwise *F*_st_ values, and the color gradient indicates the degree of differentiation, with darker shades (deep blue) representing more significant differentiation.
**Figure S5:** Variation partitioning of genetic variation explained by geographical distance and environmental factors. Each colored block represents the explanatory contribution of different components: Geography (proportion independently explained by geographical distance), Shared (jointly explained by geography and environment), Environment (proportion independently explained by environmental factors), and Residuals (unexplained variation).

## Data Availability

The raw sequence data reported in this paper have been deposited in the Genome Sequence Archive (*Genomics, Proteomics & Bioinformatics* 2025) in National Genomics Data Center (*Nucleic Acids Res* 2025), China National Center for Bioinformation/Beijing Institute of Genomics, Chinese Academy of Sciences (GSA: CRA048373) that are publicly accessible at https://ngdc.cncb.ac.cn/gsa/s/x3ObL9GG. Raw data of *Phragmites australis* transcriptome SNPs, phylogenetic relationships, functional traits, and environmental factors are available as Supporting Information.
