# Supplementary material for: Phylogenetic Constraints and Environmental Filtering Jointly Drive Adaptive Evolution in Phragmites australis : From Genetic Structure to Trait Decoupling on the Mongolian Plateau
Source: Ecol Evol. 2026 Sep 6;16(9):e74332. doi: 10.1002/ece3.74332 (PMC13546865; doi:10.1002/ece3.74332)
Supplement: Supplementary file 1 [file ECE3-16-e74332-s001.docx]

## Supporting Information

**Phylogenetic Constraints and Environmental Filtering Jointly Drive Adaptive Evolution in *Phragmites australis*: From Genetic Structure to Trait Decoupling on the Mongolian Plateau**

Rui Zhang^1,2^, Huamin Liu^1^, Shan Jiang^1^, Zhichao Xu^1^, Yunhao Wen^1^, Dang Zhenhua^1*^, Lu Wen^1^, Lixin Wang^1*^

*^1^Ministry of Education Key Laboratory of Ecology and Resource Use of the Mongolian Plateau, Inner Mongolia Key Laboratory of River and Lake Ecology, School of Ecology and Environment, Inner Mongolia University, Hohhot 010021, China*

*^2^School of Geography and Planning, Jining Normal University, Ulanqab City 012000, China*

*Correspondence: Lixin Wang: [Lxwang@imu.edu.cn](mailto:Lxwang@imu.edu.cn); Dang Zhenhua: zhdang@imu.edu.com; Tel.: 86-471-4991936

**Table S1. Geographic and climatic characteristics of the 30 *Phragmites australis* study sites**: Mean Annual Precipitation (P), Mean Annual Temperature (T), and Coordinates of the Plot.

| Site name | T (℃) | P (mm) | Latitude (°) | Longitude (°) |
| --- | --- | --- | --- | --- |
| SCD1 | 5.1 | 339 | 40.605 | 112.723 |
| SCD2 | 5.1 | 336 | 40.611 | 112.727 |
| SCD3 | 5.1 | 335 | 40.615 | 112.725 |
| WLNE1 | 0.6 | 246 | 48.369 | 117.526 |
| WLNE2 | 0.6 | 246 | 48.369 | 117.526 |
| HLG | 0.0 | 295 | 49.145 | 117.797 |
| MNAB | 0.9 | 251 | 48.619 | 117.140 |
| XHK | 0.3 | 297 | 49.317 | 117.684 |
| KLLH | 1.4 | 226 | 48.649 | 116.828 |
| BEH | 0.5 | 264 | 47.964 | 117.711 |
| DLNE | 1.5 | 352 | 43.381 | 116.659 |
| ZGST1 | 0.9 | 337 | 43.689 | 116.879 |
| ZGST2 | 0.9 | 337 | 43.689 | 116.879 |
| CGNE1 | 2.8 | 244 | 43.452 | 114.971 |
| CGNE2 | 2.8 | 244 | 43.453 | 114.972 |
| HSH1 | 6.0 | 306 | 40.616 | 110.932 |
| HSH2 | 6.0 | 311 | 40.617 | 110.931 |
| WLSH | 7.1 | 218 | 40.784 | 108.716 |
| NLH1 | 8.2 | 118 | 40.560 | 106.639 |
| NLH2 | 8.2 | 118 | 40.561 | 106.642 |
| TGNE | 8.9 | 161 | 38.737 | 105.154 |
| NRT | 9.0 | 156 | 38.626 | 104.932 |
| BDHX | 9.2 | 87 | 39.555 | 102.368 |
| BDHD | 9.2 | 90 | 39.551 | 102.366 |
| JYH | 8.3 | 43 | 42.333 | 101.268 |
| GZH1 | 9.6 | 45 | 41.367 | 102.388 |
| GZH2 | 9.7 | 45 | 41.368 | 102.409 |
| WLML1 | 6.9 | 350 | 39.587 | 109.906 |
| WLML2 | 6.9 | 360 | 39.553 | 109.977 |
| WLML3 | 6.1 | 366 | 39.702 | 110.084 |

**Table S2. Description of functional traits, units, and detailed measurement protocols.** (Cornelissen et al. 2003, Pérez-Harguindeguy et al. 2013, Kong et al. 2021, Yamauchi et al. 2021)

| Parameter | Abbreviations | unit | Calculation |
| --- | --- | --- | --- |
| Plant Height | H | cm | Measurement of plant height on-site |
| Leaf Area | LA | mm² | Plant leaf images were analyzed using ImageJ software (Version 1.52). |
| Leaf Dry Weight | LW | mg | The leaves underwent baking in an oven at 65°C for 72 hours, and we measured the dry weight using an electronic balance. |
| Leaf Thickness | LT | mm | Vernier calipers were used for on-site measurements. |
| Specific Leaf Area | SLA | mm²·mg^-1^ | SLA＝LA (mm²)/ LW (mg) |
| Leaf Dry Matter Content | LDMC | / | LDMC＝Leaf dry weight (g)/ Leaf fresh weight (g) |
| Leaf Carbon Content | LCC | mg·g^-1^ | After drying, the leaves were ground into powder, and the elemental analyzer Vario EL was employed for determination. |
| Leaf Nitrogen Content | LNC | mg·g^-1^ | After drying, the leaves were ground into powder, and the elemental analyzer Vario EL was employed for determination. |
| Net Photosynthetic Rate | A | µmol·m^-^²·s^-1^ | The net photosynthetic rate in leaves was measured in situ using the LI-6800 Advanced Photosynthesis-Fluorescence Measurement System. |
| Transpiration Rate | E | µmol·m^-^²·s^-1^ | The transpiration rate in leaves was measured in situ using the LI-6800 Advanced Photosynthesis-Fluorescence Measurement System. |
| Water Use Efficiency | WUE | / | WUE=A / E |
| Leaf Epidermis Thickness | LET | µm | Leaf midvein vascular bundles were selected and the thickness of the upper epidermis was measured several times using ImageJ software (Version 1.52) to obtain the mean value. |
| Leaf Vascular Bundle Area | LVBA | µm^2^ | ImageJ software (Version 1.52) was used to measure the area of vascular tissue in the midvein of leaves. |
| Leaf Xylem Diameter | LXD | µm | Leaf midvein vascular bundles were selected and the diameter of the xylem was measured several times using ImageJ software (Version 1.52) to obtain the mean value. |
| Leaf Phloem Diameter | LPD | µm | Leaf midvein vascular bundles were selected and the diameter of the outer phloem was measured several times using ImageJ software (Version 1.52) to obtain the mean value. |
| Leaf Upper Epidermis To Lower Epidermis Ratio | UE/LE | / | LET (µm) / lower epidermis (µm) |

**Table S3. Model selection parameters for phylogenetic trees (optimal model selected based on the lowest AICc value).**

| Model | -LnL | AIC | AICc | BIC |
| --- | --- | --- | --- | --- |
| GTR+F+ASC | 7605372 | 15211110.3 | 15211110.41 | 15213136.18 |
| GTR+F+ASC+G4 | 7405295 | 14810957.4 | 14810957.58 | 14812994.42 |
| GTR+F+ASC+R2 | 7402240 | 14804850.9 | 14804851.01 | 14806898.92 |
| GTR+F+ASC+R3 | 7396065 | 14792503.5 | 14792503.63 | 14794573.68 |
| **GTR+F+ASC+R4** | **7396002** | **14792382** | **14792382.13** | **14794474.32** |
| GTR+F+ASC+R5 | 7396556 | 14793494.7 | 14793494.83 | 14795609.16 |
| SYM+ASC+R4 | 7404675 | 14809722.3 | 14809722.46 | 14811781.44 |
| TVM+F+ASC+R4 | 7396004 | 14792383.8 | 14792383.99 | 14794465.11 |
| TVMe+ASC+R4 | 7404681 | 14809731.2 | 14809731.36 | 14811779.27 |
| TIM3+F+ASC+R4 | 7405627 | 14811627 | 14811627.2 | 14813697.25 |
| TIM3e+ASC+R4 | 7405576 | 14811520.3 | 14811520.47 | 14813557.31 |

**Table S4. Genetic diversity indices of *P. australis* populations from different geographic groups in the Mongolian Plateau.**

| Genetic Group | Expected Heterozygosity (He) | Observed Heterozygosity (Ho) | Inbreeding Coefficient (F) | Shannon_Index |
| --- | --- | --- | --- | --- |
| Cluster1 | 0.142 | 0.200 | -0.352 | 1.154 |
| Cluster2 | 0.186 | 0.242 | -0.234 | 1.077 |
| Cluster3 | 0.105 | 0.147 | -0.463 | 1.229 |
| Cluster4 | 0.196 | 0.253 | -0.213 | 1.261 |
| Cluster5 | 0.142 | 0.144 | -0.028 | 1.172 |
| Cluster6 | 0.222 | 0.314 | -0.337 | 0.911 |

**Table S5. Φ-Statistics of population genetic differentiation.**

| Statistic | Value |
| --- | --- |
| ΦST (Overall among all populations) | 0.127 |
| ΦSC (Among populations within regions) | 0.181 |
| ΦCT (Among regions) | 0.285 |

**Table S6. Results of correlation analysis between genetic distance and geographical, environmental, and phenotypic distances.**

| Analytical Method | Factor | Statistic | Value | Significance |
| --- | --- | --- | --- | --- |
| Mantel Test | Geographical Distance | r | 0.405 | p < 0.001 |
|  | Environmental Distance | r | 0.327 | p < 0.001 |
|  | Phenotypic Distance | r | 0.146 | p < 0.001 |
| Partial Mantel Test | Environmental Distance (controlling for geography) | r | 0.096 | p < 0.001 |
|  | Phenotypic Distance (controlling for geography) | r | 0.080 | p < 0.001 |
| MMRR Analysis | Geographical Distance | β | 0.315 | p < 0.001 |
|  | Environmental Distance | β | 0.117 | p < 0.001 |
|  | Phenotypic Distance | β | 0.078 | p < 0.001 |

**Fig. S1. Anatomical cross-sections of *P. australis* leaf.** (a–b) Leaf cross-sections showing the epidermis and vascular bundles (xylem and phloem).

**Fig. S2. Inference graph for selecting the ancestral population number (K-value) of the *P. australis* populations in the Mongolian Plateau.** Based on cross-entropy evaluation results from the Sparse Non-negative Matrix Factorization (SNMF) algorithm, the horizontal axis represents the assumed number of ancestral populations (K-value ranging from 1 to 10), while the vertical axis indicates the corresponding cross-entropy score. The scatter points reflect the model goodness-of-fit across different K-values, with lower cross-entropy values suggesting a more optimal population subdivision scheme.

**Fig. S3. Frequency distribution of pairwise genetic differentiation coefficient (Fst) among *P. australis* populations in the Mongolian Plateau.** The histogram displays the distribution of Fst values calculated between all pairs of sampling populations. The horizontal axis represents the calculated Fst value, and the vertical axis represents the frequency (Count). The text above the graph indicates the mean pairwise Fst value for all samples.

**Fig. S4. Heatmap of pairwise Fst differentiation among the six major genetic groups of *P. australis* populations in the Mongolian Plateau.** The matrix illustrates the genetic differentiation between different genetic groups (Cluster 1–6). The values within the grids represent pairwise Fst values, and the color gradient indicates the degree of differentiation, with darker shades (deep blue) representing more significant differentiation.

**Fig. S5. Variation partitioning of genetic variation explained by geographical distance and environmental factors.** Each colored block represents the explanatory contribution of different components: Geography (proportion independently explained by geographical distance), Shared (jointly explained by geography and environment), Environment (proportion independently explained by environmental factors), and Residuals (unexplained variation).

## References

Cornelissen, J. H. C., S. Lavorel, E. Garnier, S. Díaz, N. Buchmann, D. E. Gurvich, P. B. Reich, H. T. Steege, H. D. Morgan, M. G. A. V. D. Heijden, J. G. Pausas, and H. Poorter. 2003. A handbook of protocols for standardised and easy measurement of plant functional traits worldwide. Australian Journal of Botany 51:335.

Kong, D., J. Wang, O. J. Valverde-Barrantes, and P. Kardol. 2021. A framework to assess the carbon supply–consumption balance in plant roots. New Phytologist 229:659–664.

Pérez-Harguindeguy, N., S. Díaz, E. Garnier, S. Lavorel, H. Poorter, P. Jaureguiberry, M. S. Bret-Harte, W. K. Cornwell, J. M. Craine, D. E. Gurvich, C. Urcelay, E. J. Veneklaas, P. B. Reich, L. Poorter, I. J. Wright, P. Ray, L. Enrico, J. G. Pausas, A. C. De Vos, N. Buchmann, G. Funes, F. Quétier, J. G. Hodgson, K. Thompson, H. D. Morgan, H. Ter Steege, L. Sack, B. Blonder, P. Poschlod, M. V. Vaieretti, G. Conti, A. C. Staver, S. Aquino, and J. H. C. Cornelissen. 2013. New handbook for standardised measurement of plant functional traits worldwide. Australian Journal of Botany 61:167.

Yamauchi, T., O. Pedersen, M. Nakazono, and N. Tsutsumi. 2021. Key root traits of poaceae for adaptation to soil water gradients. New Phytologist 229:3133–3140.
